# Arterial Junctional Hemostasis without Compression: Evaluation of Visco-liquid Hemostats in Male Swine^✰^^✰^

**DOI:** 10.1016/j.bbiosy.2025.100111

**Published:** 2025-04-11

**Authors:** Michelle A. Tucci, Joseph D. Lichtenhan, Hamad A. Benghuzzi, Drew A. Hildebrandt

**Affiliations:** a Anesthesiology, University of Mississippi Medical Center, 2500N. State Street, Jackson, MS, 39216, USA; b Hybrid Plastics, Inc., 55 WL Runnels Industrial Drive, Hattiesburg, MS, 39401, USA; c Biology Department, Jackson State University, 1400J.R. Lynch Street, P.O. Box 18540, Jackson, MS, 39217, USA; d Surgery, University of Mississippi Medical Center, 2500N. State Street, Jackson, MS, 39216, USA

**Keywords:** Trimethylpentyl polysilsesquioxane, Poss, Hemostasis, Chitin, Kaolin, Noncompressive

## Abstract

Trimethylpentyl polysilsesquioxane (POSS) gels containing kaolin and chitin promote clotting and stabilize and strengthen thrombi in vitro, and may offer a potential solution for treating non-compressible wounds. This study was designed to determine if the gels could stop bleeding in a junctional arterial hemorrhage model. Anesthetized male swine were instrumented for systemic arterial pressure (MAP) measurement and saline infusion. A femoral artery was punctured (2 × 6 mm), allowed to bleed freely for 45 s, and then either QuikClot gauze bandage (QC; *n* = 7), POSS-Kaolin (PK) or POSS-Chitin (PC) (40 ml; *n* = 7/group) applied with no external compression. Blood loss (BL) at 60 min post-treatment was greater in QC (1166±79 ml) than PK (188±74 ml; *p* < 0.0001) or PC (523±116 ml; *p* = 0.0001); BL in PC was greater than in PK (*p* = 0.03). Total BL (180 min) was higher in QC (1210±93 ml) than PK (475±85 ml, *p* < 0.001) or PC (632±133 ml; *p* = 0.002) and in PC vs PK (*p* = 0.008). Time to clot was not different between PK (3 ± 1) or PC (10±3 min), but was longer in QC (44±9 min) than PK or PC (*p* < 0.0001 vs PK, *p* < 0.0003 vs PC). MAP fell 40±3 mmHg in QC by 10 min post-injury (*p* < 0.0003), and remained below control. PC MAP fell 41±5 mmHg, but returned to control, and MAP did not change in PK. POSS in combination with kaolin or chitin provided hemorrhage control and systemic hemodynamic stability without compression. These results support the treatment concept that this new approach to hemostasis can be efficacious in treating non-compressible trauma wounds.

## Highlights

• Traumatic non-compressible hemorrhage causes considerable preventable deaths.
• No current effective treatment for non-compressible wounds with brisk venous or arterial blood flow.
• Trimethylpentyl polysilsesquioxane viscogels (POSS) yielded hemostasis without compression.
• POSS can be efficacious in treating non-compressible trauma wounds.

## Graphical abstract

**Figure:**
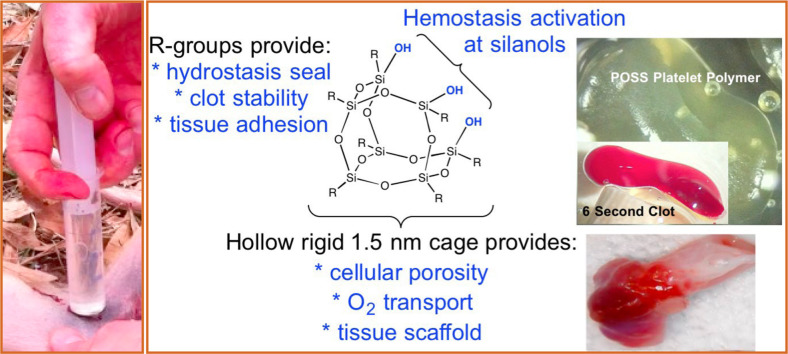

## 1 Introduction

Non-compressible hemorrhage causes a large percentage of preventable deaths on the battlefield and in civilian life [1,2]; thus, effective pre-hospital treatment is essential for reducing this morbidity and mortality [3]. Despite development of numerous hemostats, no product currently is able to produce effective hemostasis in wounds with brisk venous or arterial blood flow [4,5]. All hemostatic products of which we are aware either cause compression (e.g. tourniquets, ResQFoam™, XSTAT®); or require that blood flow be slowed considerably by other means such as compression or surgical closure for effective treatment (e.g. Celox-A™, Surgicel^TM^; see supplement document 1 for a more complete list of hemostatic products); in most cases requiring direct access to the wound, thus leading one group to state “…the lack of clear superiority of any agent suggests that contemporary hemostatic dressing technology has potentially reached a plateau for efficacy.” [6] Although hemostatic dressings, devices such as QuikClot®, ResQFoam,™ and various tourniquets and other devices have proven useful [7], they are not suitable for extended care in the pre-hospital setting because they block visual access of the wound and are subject to rebleed upon patient transport/movement [8].

Compression at the point of injury has long been a standard of treatment. To be effective, the direct pressure of compression must slow the bleeding sufficiently to enable the coagulation cascade to catch-up and create a thrombus, which requires a suitable surface against which clotting components can adhere and build a thrombus. Interestingly, comparative studies utilizing coagulation enhanced hemostatic devices all appear “similar” in their effectiveness [9]. This finding perhaps can be explained by an overwhelming effect of compression and a secondary effect of the coagulation surface. Hemostatic treatments that do not require compression would be advantageous, primarily because they have the potential to work in situations where adequate compressive hemostasis is not feasible.

One technology with promise for treating non-compressible hemorrhage is trimethylpentyl polysilsesquioxane viscogels (POSS®). They have the ability to prevent fluid loss immediately while simultaneously providing a temporary surface against which a thrombus can form [10]; require no mixing or special handling, and are applied to a wound via a warfighter doypack or flushing syringe; thus, they have the potential for treating difficult-to-access wounds.

Visco-liquid hemostat formulations based upon POSS promote clotting and stabilize and strengthen thrombi from junctional arteries in vitro [10]. In particular, silanol (Si-OH) containing POSS activates the clotting cascade in a manner similar to that of kaolin. Furthermore, the combination of POSS silanols with known clotting agents such as kaolin and chitin show enhanced viscoelasticity, which aids in sealing the wound against gel displacement and fluid loss by pulsating blood flows and tissue exudation.

The effectiveness of these gels has not been evaluated in vivo, especially in models of non-compressible vascular injury. Therefore, the aim of this study was to examine their hemostatic efficacy in a swine femoral injury model without internal or external compression. This deliberate departure from standard of care was intended to provide insights useful toward the development of a rapidly deployable self/buddy method of hemorrhagic care under austere conditions.

## 2 Methods

### 2.1 Assurances

The University of Mississippi Medical Center Institutional Animal Care and Use Committee, and the United States Army Medical Research and Development Command Animal Care and Use Office reviewed and approved all the experiments in this study. The Association for Assessment and Accreditation of Laboratory Animal Care accredits the UMMC facilities where these experiments were conducted. In conducting this research, the investigators adhered to the Animal Welfare Act Regulations and other Federal statutes relating to animals and experiments involving animals and the principles set forth in the current version of the *Guide for the Care and Use of Laboratory Animals, National Research Council* [11]. This report follows the ARRIVE guidelines for reporting animal research [12].

### 2.2 Animal preparation

Male adult pigs (*n* = 27) were obtained from Valley Brook Farms, Madison, Georgia, and were held for a minimum of 5 days to allow for acclimatization and for observation by UMMC veterinarians. During this time, they had free access to food and water; they were denied food starting ∼17 h prior to being anesthetized. After premedication with an intramuscular cocktail of atropine (0.025 mg/kg), dexmeditomidine (0.08 mg/kg), and butorphanol (0.2 mg/kg), anesthesia was induced with ketamine (10 mg/kg i.m.). Following orotracheal intubation they either were allowed to breathe room air or were ventilated as needed to maintain a PO_2_ of at least 95 % and an end-tidal CO_2_ of 40–45 %. They initially were maintained on isoflurane at 3 %; the isoflurane was reduced to 2 % following surgery and for the duration of the experiment. The left carotid artery and internal jugular vein were exposed via cutdown, and a Millar catheter (Millar, Inc., Houston, TX) inserted into the artery for continuous measurement of MAP and heart rate (HR) using a PowerLab data acquisition system and LabChart software (ADInstruments, Inc., Colorado Springs, CO). A jugular vein was cannulated for infusion of sterile isotonic saline at ∼6 ml/kg/hour for the duration of the procedure and for administration of resuscitation fluids. The left femoral artery was exposed via an inguinal incision and the overlying adductor muscle removed to provide unobstructed access to the artery. Two silk ties, one proximal and one distal, were placed underneath the artery for retraction, otherwise the artery was left undisturbed. A lateral channel was produced by dissecting through the fascia on the anterolateral side of the left leg to an ∼10 cm exit incision through the skin and muscle on the left flank of the pig. This lateral channel allowed for a secondary route for blood to exit the vascular wound site thus reducing the ability of any hydrostatic pressure from blood collecting in the inguinal pocket to contribute to hemostasis.

### 2.3 POSS polymer gels

POSS [13] was provided by Hybrid Plastics Inc. at *a* < 99 % devolatilized purity level, thermally sterilized by falling film distillation at 120 °C (@10^–6^ torr) over 4 hr [14].

In order to increase coagulation ability and durability of the gel seal against displacement by blood, POSS was mixed with either kaolin (PK, 87.5:22.5 wt %) or chitin (PC, 90:10 wt %). Both substances were used as received from Sigma Aldrich (St. Louis, MO, USA). The mixing was accomplished using a high-shear mixer (Silverson, East Longmeadow, MA, USA, 5000 rpm, 5 min, 90 °C) until visual homogeneity and steady-state torque were observed [14].

### 2.4 Experimental protocol

After 30 min of recovery time and 15 min of control data acquisition, the femoral artery ties were retracted to occlude flow, a 2 × 6 mm hole punched in the artery (Scanlon International, Inc., Saint Paul, MN, USA), and the ties removed completely. The wound was allowed to bleed freely for 45 s and then one of the following treatments applied in random order (simple randomization using Microsoft Excel (Rand) function, not blinded): 1. 40 ml PK, syringed into the wound cavity (*n* = 7); 2. 40 ml PC, syringed into the wound cavity (*n* = 7); or 3. QuikClot (QC) combat bandage (Z-Medica, LLC, Wallingford, CT, USA) held just above the wound so that blood could contact the bandage but no pressure applied to the artery (*n* = 7). It should be noted that this is not the recommended application method for QC. We applied it in this manner to mimic a wound that could not be packed tightly enough to provide compression of the artery. Although not providing compressive hemostasis, this allowed blood squirting out of the artery to contact the bandage, interact with the kaolin bandage, and fall back into the wound, thus helping to enhance hemostasis. Three minutes after application the QC was removed. By this time blood no longer was contacting the bandage. Ten minutes after wounding, 500 ml 6 % hetastarch in isotonic saline (Hextend, Hospira, Inc., Lake Forest, IL, USA) was infused i.v. over 30 min. Blood was collected via aspiration and blotting with pre-weighed laparotomy pads or gauze and the amount of blood determined gravimetrically. Blood was collected during the 45-second pre-treatment period and for three consecutive 60-minute periods following treatment application. Every 30 min following treatment, the left leg was extended and retracted vigorously 20 times to test the integrity of the clot against rebleeding and to simulate patient movement during evacuation. At 180 min post-treatment the pigs were euthanized, the POSS carefully removed by wiping with gauze, the wounded section of the artery clot visualized, and that section of artery—including any clot—was excised for visual determination of the patency of the artery.

### 2.5 Preliminary experiments

**Fig. 1:**
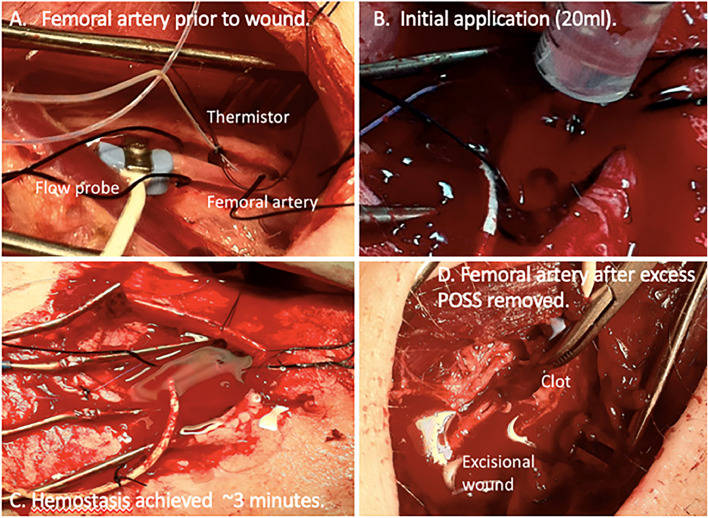
(a) Instrumentation of injury site during preliminary protocol development; (b) application of gel; (c) hemostasis without compression; (d) exposure of thrombus after gel removal.

Preliminary experiments were conducted utilizing six male swine to finalize a protocol. In these animals, in addition to the preparation outlined above, ultrasonic perivascular flow probes (Transonic Systems, Inc., Ithaca, NY, USA) were placed on the femoral artery distal to the wound site in order to monitor blood flow past the wound (Fig. 1). An implantable flexible microprobe (Physitemp Instruments LLC, Clifton, NJ, USA) was sutured to muscle adjacent to the femoral artery. Temperature at the wound site was recorded continuously throughout the procedure. These preliminary animals were treated only with the POSS gel.

### 2.6 Data analysis

MAP and HR and, in preliminary experiments, wound-site temperature, were sampled continuously at 100 Hz and then analyzed in 5-minute blocks. The time to reach hemostasis was made visually by constant examination of the wound (top and lateral channels) for active bleeding by 2 or 3 observers (non-blinded). The time-to-hemostasis was determined from the time treatment was applied until bleeding initially stopped. It does not include any rebleeding and reclotting events. Two-way Analysis of Variance for Repeated Measures was used for initial analysis of the MAP and HR data, followed by Dunnett's test for data within a group. Between-group data for each time point were analyzed with ANOVA followed by Tukey's Multiple Comparison Test. Time-to-clot data also were analyzed using ANOVA and Tukey's. All statistical tests were performed using GraphPad Prism software (GraphPad Software, San Diego, CA, USA). All data are presented as mean ± SEM, and data values were considered different at an alpha level of 0.05. No large-language models of any kind were used to write any part of this manuscript.

## 3 Results

No animals were excluded from this study. There were no differences between groups in weight, or baseline MAP or HR (Supplementary material Table 1).

**Fig. 2:**
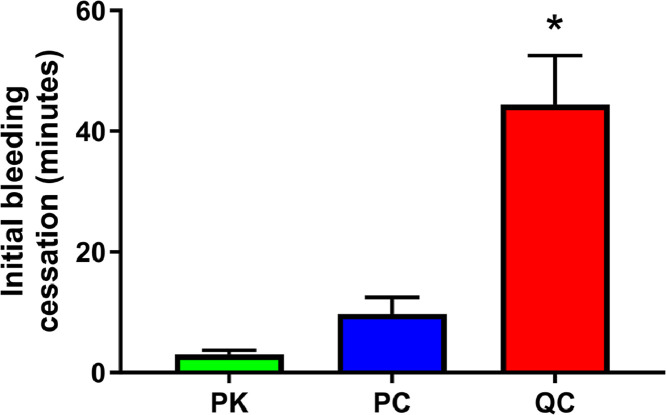
Time to initial hemostasis in minutes following treatment of hemorrhage with either POSS-kaolin (PK, *n* = 7), POSS-chitin (PC, *n* = 7) or QuikClot combat gauze (QC; without compression, *n* = 7). *p < 0.05 when compared with either PK or PC.

There was no significant difference in duration from start of treatment to initial cessation of bleeding between the PC group (3 ± 1 min) and the PK animals (10 ± 3 min; Fig. 2). As anticipated, the use of QC in a non-compressive manner limited its ability to provide a sufficiently penetrating surface to effectively concentrate and dry the wound to the point of coagulation despite QC being in contact with blood filling the wound. Although all of the QC pigs eventually did cease to bleed, comparably, the time to the initial cessation of bleeding was substantially longer in QC than in either PK or PC (44 ± 9 min; *p* < 0.0001 vs PK; *p* < 0.0003 vs PC). Furthermore, none of the QC pigs were able to maintain this hemostasis for the duration of the entire observation period. Instead, in the QC animals, spontaneous bleeding occurred periodically throughout the post-clot period, generally following increases in MAP.

**Fig. 3:**
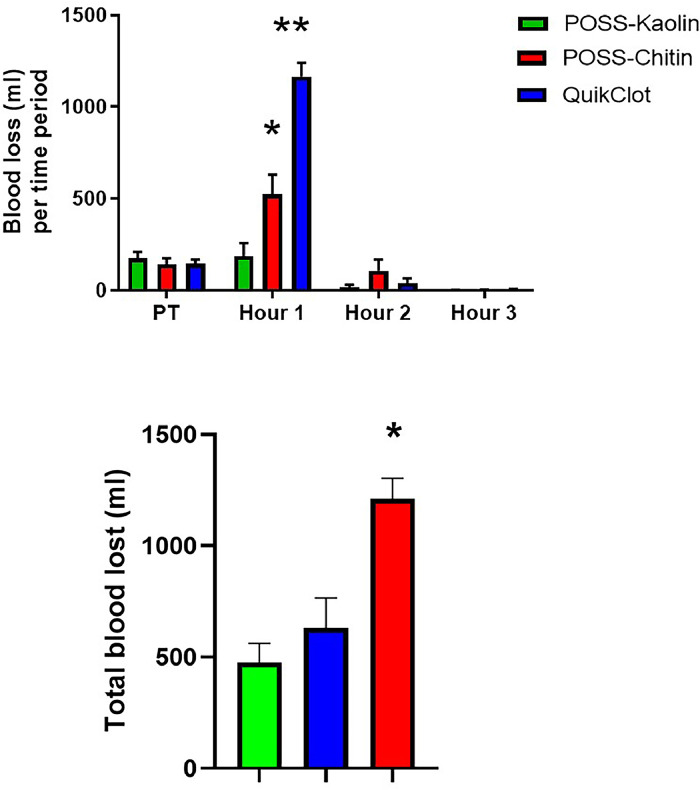
Top Panel: Total amount of blood lost following hemorrhage (Pre-treatment free bleed period=45 s), and treatment (Hours 1, 2. and 3) for POSS-kaolin (*n* = 7), POSS-chitin (*n* = 7) or QuikClot combat gauze (without compression; *n* = 7). Blood was collected during the 45-second pre-treatment period and for three consecutive 60-minute periods following treatment application. *p < 0.05 when compared with POSS-kaolin. **p < 0.05 when compared with either POSS-kaolin or POSS-chitin.

Pre-treatment blood loss following injury was not different among the groups (186 ± 33 ml in PK; 141 ± 38 in PC; 143 ± 27 ml in QC; Fig. 3). However, because of the longer time to the initial cessation of bleeding in the QC group, over the first hour post-treatment QC pigs lost substantially more blood (1166 ± 79 ml) than either PK (188 ± 74 ml; *p* < 0.0001) or PC (523 ± 116 ml; *p* = 0.0001). Blood loss in PC was significantly greater than in PK during this time (*p* = 0.03), also reflecting the slight difference in clotting times. During the next two hours, blood loss was negligible and not different among the groups. Total blood lost during the entire post-treatment period was significantly higher in QC (1210 ± 93 ml) than in either of the other groups (PK = 475 ± 85 ml, *p* < 0.001; PC = 632 ± 133 ml; *p* = 0.002; *p* = 0.008 PK vs PC).

Bottom Panel: Total amount of blood lost during entire 180-minute post-treatment period for POSS-kaolin (*n* = 7), POSS-chitin (*n* = 7) or QuikClot combat gauze (without compression, *n* = 7). *p < 0.05 when compared with either POSS-kaolin or POSS-chitin.

**Fig. 4:**
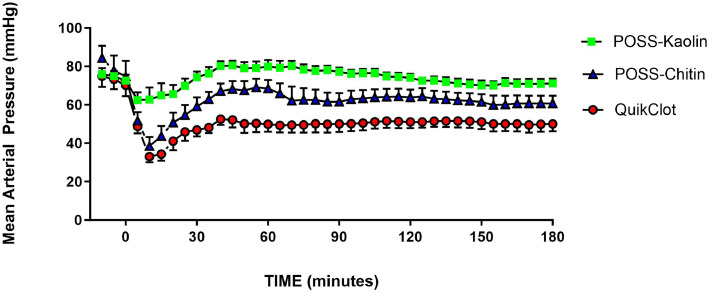
MAP prior to and following hemorrhage for POSS-kaolin (*n* = 7), POSS-chitin (*n* = 7) and QuikClot combat gauze (without compression; *n* = 7). Each symbol represents 5-minute data block. Treatment was initiated 45 s following production of the wound.

In response to the loss of blood, MAP fell significantly post-wound in QC (nadir was at a decrease of 40 ± 3 mmHg at 10 min post-bleeding, *p* < 0.0003; Fig. 4). Furthermore, MAP remained below control values during the remainder of experiment in QC, and below the 65 mmHg MAP generally accepted to be the point at which rebleeding occurs in this model [15]. Blood pressure did fluctuate in individual animals in this group in that the MAP would rise to near 65 mmHg, then bleeding would start and MAP would fall until bleeding stopped. This continued generally for the duration of the post-treatment period. In contrast to the changes in MAP in QC animals, MAP did not change significantly in the PK pigs following injury and remained significantly above the MAP seen in QC animals for the entire experiment post-hemorrhage, and above the 65 mmHg rebleed pressure. Changes in MAP for PC were intermediate (nadir at 41 ± 5 mmHg at 10 min post-bleed; *p* = 0.015) with MAP returning to a level between PK and QC and also not significantly lower than control values, at or above 65 mm. Although there was a general trend for HR to decrease with time in each group, HR was statistically unchanged from control in any group for the duration of the experiment and was not different among the groups.

Vigorous leg movements failed to dislodge the clot or cause rebleeding in any of the POSS pigs but always caused further bleeding in the QC animals in which bleeding had previously been stopped even though there were no detectable changes in MAP in any of the animals as a result of this movement.

**Fig. 5:**
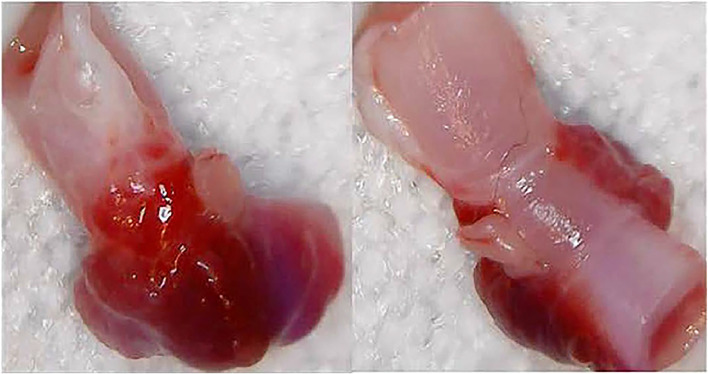
Top and bottom views of a representative femoral artery section containing the gel-induced thrombus at point of injury. The thrombus (red) adheres to the wound, but the gel is easily removed given visual access to the wound.

Finally, Fig. 5 illustrates a typical femoral artery clot after removal of that section of artery following the termination of the experiment. In none of the arteries examined did the clot interfere with the lumen of the artery, nor was there any evidence the gels had invaded the artery following application.

These results are complimented by two findings from our preliminary protocol development work involving six swine.

First, in none of the POSS-treated animals did the temperature rise in tissue immediately adjacent to the injury following treatment (37 ± 0.4 control vs. 36 ± 0.8 °C for the first 15 min post-treatment; *n* = 6). Although we cannot rule out localized tissue effects, because there was no overt hyperthermic reaction to the application of gel we did not feel it necessary to continue to measure tissue temperatures at the wound site in the remaining experiments.

Second, we also were able to detect pulsatile blood flow in the femoral artery distal to the wound following injury and treatment in all of the animals. Measured blood flow was 118 ± 15 ml/min during control and fell to 42 ± 28 ml/min during the 15 min post-treatment.

## 4 Discussion

This study provides data that hemostasis upon POSS- treatment of brisk arterial hemorrhage without compression is possible; thus, viscogels may represent a promising approach for pre-hospital treatment of hemorrhage in warfighters or civilian trauma principally because the gels could reduce the need for external compression to be applied to the injury. Furthermore, the maintenance of arterial blood pressure at or near the pre-hemorrhage value in the POSS-treated animals is important in that it means that blood flow to vital tissues and organs should be preserved following this treatment. The ability of POSS gels to enable limb mobilization without rebleeding is significant in that they would prove useful in situations where hands-on compression might be necessary, insomuch as they would mitigate the need for an extra set of hands to provide the compression, or to maintain the compression during long periods of time or during movement of the injured person. This finding suggests that POSS gels may be of value for austere transports. Finally, although very preliminary, the observation that pulsatile blood flow to limb distal to the wound following treatment is encouraging in that, unlike tourniquets, for example, POSS gels may provide hemostasis while minimizing downstream ischemia.

In this study, the wound site was accessible, but the exact point at which the artery was wounded was obscured by blood, so the gel was applied blindly to the wound area and allowed to flow and fill into the wound site, much as would be the case in wounds treated in the field. Despite the fact the gels flow, adequate POSS remained over the arterial puncture to promote hemostasis while not occluding the treated arteries. This flow property of POSS could prove efficacious for treating wounds that cannot be easily visualized, or more complex wounds, such as crushed liver or lacerated lung. In fact, POSS viscoelastic gels have provided hemostasis in an experimental model of the latter [16]

The thrombi formed through contact with POSS viscoelastic gels were not easily dislodged, either by movement of the limb or wiping with gauze at the end of the experiment. However, the gels were easily removed from the wounds by wiping with gauze, which allowed clear access to visualize the wounded artery at the end of the experiment. This ease of removal would be important during later treatment at an aid facility. Furthermore, we did not observe any negative interactions with tissue in our 3-hour acute study. This observation agrees with results of previous experiments with the gels and cells in culture [10], or skin wound healing, [[17], [18], [19]] that POSS has no adverse effects when applied, even when left on cells in culture for or on skin wounds for up to 21 days. This observation is important in that it means that does not have to be removed immediately, if at all, and can remain in the wound for as long as necessary for proper treatment.

The fact that leg movement did not dislodge POSS-induced clots in this study indicates that POSS treatment could minimize the need to immobilize a wound to prevent dislodgement of the thrombus, which would make it more useful in a field setting, especially in an austere environment.

There are some limitations with this study that should be considered. First, these studies were conducted in a model that provided easy visual and physical access to the artery. Of course, in real situations, compression easily would be applied to this type of wound to help with hemostasis in conjunction with use of other treatments such as QC. We chose this model as the first iteration in hemostasis studies involving POSS gels because the degree of hemorrhage induced was easily controlled; measurement of blood loss more easily quantified; and the cessation of bleeding more easily determined. We recognize that these results may not be applicable to other hemorrhagic wounds, but it must be kept in mind that this was the first study of this type and investigations of other types of wounds are needed. The fact that we chose this open model does not detract from our findings that POSS gels provided hemostasis without additional treatments such as compression. Further, one advantage of this model is that the treatment was effective in a situation where there was no additional compression provided by surrounding tissues, or the hydrostatic pressure from a column of POSS as one could find in a more enclosed, deeper wound site.

A second limitation could be the design of our control group. We wanted to use a control that did not produce any compression of the wound, which would have rendered such a control useless in comparing with our POSS groups. However, we also wanted to provide some semblance of attempted treatment instead of just allowing the artery to bleed freely, which also would not have been a good comparison to our POSS groups. Finally, we wanted to use a device that commonly is used in these situations. Thus, QC was chosen as a control device because of its successful and wide usage in the military and allowance for over-the-counter use. The active agent in QC is kaolin and this same active agent is present in the PK formulation, thus allowing a better direct comparison to PK. We do recognize the indication-for-use of QC requires that it be utilized with compression and thus we did not apply the device as recommended. It is postulated this finding with QC may be similar to what could happen for a QC bandage that was unable to be packed deeply enough and compressively held in place under austere conditions. In this situation, the medic or first responder still would pack the wound as best they could, even if it means the impregnated gauze did not physically contact the wounded artery [20].

A third limitation is that the cessation of bleeding assessments were unblinded. Ideally one would prefer all the assessors be blinded to the treatment. However, it was exceedingly clear just looking at the wound as to what treatment was applied, so there realistically was no way to blind the assessors completely. Further, the assessment was binary—was it bleeding or not bleeding—instead of having gradations of bleeding that require a judgment to be made on the “type” of bleeding, so the need for blinded viewers was minimized. Thus, while this could potentially be a factor, we do not think it had any influence on the impact of this study.

A fourth factor that may be important in the interpretation of these results and their application to the overall population is that all the animals used in this experiment were male. There is a sex difference in blood clotting in humans [20], however, females are hypercoagulable compared with males, so if there is any bias based on sex in this study, one would presume it should have acted against a favorable response to the POSS treatments, and thus advocates for the use of POSS in both sexes.

Other minor limitations are that this study tested POSS in only one type of wound; further studies are necessary to determine the scope and range of non-compressive gel treatments. Also, quantitative data on blood flow distal to the wound following POSS treatment are lacking. Although our preliminary experiments, and the fact that in none of the POSS animals was the arterial lumen blocked, strongly suggest that distal blood flow was maintained, direct measurements of blood flow distal to the wound need to be made. Such data could prove important in providing further data as to whether the gel does enter the lumen of the vessel, and to what extent it has a negative effect on downstream blood flow.

Finally, a perfect hemostat does not exist given the wide variances in wounds and physiology. Therefore, tourniquets and gauze will always have their role. However, they may be utilized in conjunction with other hemostats. For example, it can be envisioned that a tourniquet may be necessary as an initial stop-gap and could later be slowly released after the application of a visco-gel to a terminated appendage. The advantage of such a transitional treatment would be retainment of blood flow to as much of the appendage as possible thus reducing the prospect of limb amputation.

## 5 Conclusion

Trimethylpentyl polysilsesquioxane viscoelastic gels are, to our knowledge, the only treatment option for arterial hemorrhage that does not require, nor induce, compression in order to stop bleeding, at least as can be indicated from this one seminal study. The key attributes contributing to the effectiveness of the gels include the viscoelastic characteristic, the adhesive characteristic, and the surface characteristic of the gel. Further studies on different types of wounds, more severe wounds, and in coagulopathic conditions are needed to determine the scope and range of their utility in civilian and battlefield polytrauma. Some of these studies already are underway and others along these lines are planned for future endeavors. Financial support was provided by US Army Medical Research and Development Command Contract No. W81XWH-17-C-0184 (J.D.L, P.I.) and National Science Foundation EPSCOR NSF-EPS-0903,787 (D.A.H., P.I.).

## CRediT authorship contribution statement

**Michelle A. Tucci:** Conceptualization, Methodology, Investigation, Writing – review & editing. **Joseph D. Lichtenhan:** Conceptualization, Funding acquisition, Investigation, Methodology, Writing – review & editing. **Hamad A. Benghuzzi:** Conceptualization, Methodology, Writing – review & editing. **Drew A. Hildebrandt:** Conceptualization, Data curation, Formal analysis, Funding acquisition, Investigation, Methodology, Project administration, Writing – original draft, Writing – review & editing.

## Declaration of competing interest

The authors declare the following financial interests/personal relationships which may be considered as potential competing interests: Michelle A. Tucci, Ph.D. reports financial support was provided by Hybrid Plastics, Pass-through grant from US Army Medical Research and Development Command under Contract No. W81XWH-17-C-0184. Joseph D. Lichtenhan, Ph.D. reports financial support was provided by US Army Medical Research and Development Command under Contract No. W81XWH-17-C-0184. Hamad A. Benghuzzi, Ph.D. reports financial support was provided by Hybrid Plastics, Pass-through grant from US Army Medical Research and Development Command under Contract No. W81XWH-17-C-0184. Drew A. Hildebrandt, Ph.D. reports financial support was provided by Hybrid Plastics, Pass-through grant from US Army Medical Research and Development Command under Contract No. W81XWH-17-C-0184. Drew A. Hildebrandt, Ph.D. reports financial support was provided by National Science Foundation EPSCOR NSF-EPS-0903,787. Joseph D. Lichtenhan, Ph.D. has patent #U.S. Patent 7572,872 “Biomimetic Materials Comprising Polyhedral Oligomeric Silsesquioxanes issued to Joseph D. Lichtenhan, Ph.D. Joseph D. Lichtenhan, Ph.D. has patent #Pending Application 17,599,369 “POSS Viscoelastic Hemostatic Agents. pending to Joseph D. Lichtenhan, Ph.D. If there are other authors, they declare that they have no known competing financial interests or personal relationships that could have appeared to influence the work reported in this paper.

## Data Availability

Data will be made available on request.

## Appendix Supplementary materials

Image, application 1

Image, application 2

Image, application 3
