# Supplementary material for: Arterial Junctional Hemostasis without Compression: Evaluation of Visco-liquid Hemostats in Male Swine✰
Source: Biomater Biosyst. 2025 Apr 11;18:100111. doi: 10.1016/j.bbiosy.2025.100111 (PMC12135374; doi:10.1016/j.bbiosy.2025.100111)
Supplement: Supplementary file 1 [file mmc1.docx]

Skylar C. Rodgers, Kristen T. Carter, Deepti Patki, Robert C. O’Brien, and Matthew E. Kutcher. Thromboelastography-Based Evaluation of Gender-Associated Hypercoagulability. The American Surgeon 2022; [88(11](https://journals.sagepub.com/toc/asua/88/11)): 2619-2625.
