# Supplementary material for: Arterial Junctional Hemostasis without Compression: Evaluation of Visco-liquid Hemostats in Male Swine✰
Source: Biomater Biosyst. 2025 Apr 11;18:100111. doi: 10.1016/j.bbiosy.2025.100111 (PMC12135374; doi:10.1016/j.bbiosy.2025.100111)
Supplement: Supplementary file 2 [file mmc2.docx]

Supplement 1.

Directions and contraindications for use of topical hemostats

All information is directly from the manufacturer’s web site or package inserts. The following are direct quotes from the manufacturer.

Arista AH ^TM^ “Arista AH is indicated in surgical procedures as an adjunctive hemostatic device to assist when control of capillary, venous and arteriolar bleeding by pressure.... are ineffective.”

Avitene ^TM^ “Should not be used in instances of pumping arterial hemorrhage.”

Celox ^TM^ -A “Pour granules on to the area of the wound. Pack – cover the granules with gauze and fill any remaining cavity with gauze. Press. Compress the wound to push the Celox into place and stop blood flow for a short period while the Celox clot develops and strengthens.” “Not indicated for junctional wounds not amenable to pressure.”

Evarrest^®^ “Is a fibrin sealant patch indicated for use with manual compression as an adjunct to hemostasis in adult patients undergoing surgery.....”

Evicel^®^ “Contraindicated for brisk arterial bleeding.”

Fibrillar^®^ Surgicel Fibrillar is a veterinary version of Surgicel and has the same directions for use as that product.

Gelfoam^®^ “A single piece of Gelfoam should be manually applied to the bleeding site, and held in place with moderate pressure until hemostasis results.”

Rapid-Seal™ “Apply enough gel to completely cover the surface area of the wound. Using a gauze pad, press and hold for approximately 30-60 seconds. Gel controls bleeding by adhering to wound and sealing it.”

ResQFoam ^TM^ “Treatment consists of an injection of two liquid polymers that react upon combining to create a foam that expands rapidly through actively flowing blood to compress the injury and control bleeding.”

Seal-It^®^ Is a veterinary version of Rapid-Seal™. “Apply enough gel to completely cover the surface area of the wound. Using a gauze pad, press and hold for approximately 30-60 seconds. Gel controls bleeding by adhering to wound and sealing it.”

Surgicel^®^ “Designed to control continuous oozing bleeding on broad surfaces. Use only as much Surgicel as necessary for hemostasis, holding it firmly in place until the bleeding stops.”

SurgiFlo^TM^ “Should not be used in instances of pumping arterial hemorrhage.”

SurgiMend^®^ “Intended for implantation to reinforce soft tissue where weakness exists and for the surgical repair of damaged or ruptured soft tissue membranes.”

Tachosil^®^ “Not for use in place of sutures....in treatment of major arterial or venous bleeding.”

Thrombi-Gel^®^ “Place the wetted Thrombi-Gel directly over the source of the bleeding and apply adjunct manual compression until hemostasis is achieved.”

Tisseel “Do not use Tisseal for treatment of severe or brisk arterial or venous bleeding.”

VetiGel^®^ “Vetigel^®^ is indicated as an adjunctive hemostatic device to assist when control of bleeding by pressure, ligature and other conventional procedures in ineffective or impractical.”

Vistaseal ^TM^ “Do not use for the treatment of severe or brisk arterial bleeding.”
