# Supplementary material for: Arterial Junctional Hemostasis without Compression: Evaluation of Visco-liquid Hemostats in Male Swine✰
Source: Biomater Biosyst. 2025 Apr 11;18:100111. doi: 10.1016/j.bbiosy.2025.100111 (PMC12135374; doi:10.1016/j.bbiosy.2025.100111)
Supplement: Supplementary file 3 [file mmc3.docx]

**Supplement Table 1**. Mean pig weight and baseline systemic hemodynamic variables.

|  | Weight (kg) | MAP (mmHg) | HR (beats/min) |
| --- | --- | --- | --- |
| PK | 43 ± 2 | 74 ± 3 | 95 ± 6 |
| PC | 41 ± 1 | 79 ± 8 | 100 ± 5 |
| QC | 42 ± 2 | 73 ± 6 | 89 ± 3 |
| p-value | p = 0.493 | p = 0.698 | p = 0.253 |

Values are mean ± standard error. Baseline hemodynamic data are mean of three 5-minute data collections prior to injury for each animal. MAP = mean systemic arterial blood pressure; HR = heart rate; significance represented as p-values.
